# Response to Sunjaya AF, Sunjaya AP, “Pooled Testing for Expanding COVID-19 Mass Surveillance”

**DOI:** 10.1017/dmp.2020.454

**Published:** 2020-11-19

**Authors:** Wan Ki Chow, Cheuk Lun Chow

**Affiliations:** 1 Department of Building Services Engineering, The Hong Kong Polytechnic University, Hong Kong, China; 2 Department of Architecture and Civil Engineering, City University of Hong Kong, Hong Kong, China

**Keywords:** pool tests, mathematical support

We read the above article^1^ with great interest and would like to add a key point on the “optimal” pool size *n* in detecting severe acute respiratory syndrome coronavirus 2 (SARS-CoV-2). The maximum pooled size can be up to 64, as reported.^1^ For an observed population infection rate *θ* tested earlier, testing a group size of *m* people waiting to be tested, with *m* = 1/θ, is likely to have 1 positive detection result. The total number of tests *L* for this group of *m* people with a pooled size *n* can then be expressed by 2 terms:

- The number of tests with samples pooled *m*/*n*, and
- The additional tests *n* required on the pool having a positive detection.(1)
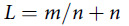

The minimum value of *L* can be found by differentiating *L* w.r.t. *n* and setting *dL/dn* = 0, yielding *n = m* ^1/2^. The minimum value of *L* is thus 2*n*.

Thus, for an observed infection rate of θ = 0.01, *m* = 100. If people to be tested are divided into groups of 100, the optimal pooled size 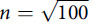 or 10. The minimum value of *L* is only 20, instead of doing 100 tests for all individual samples.

For a large population, people can be grouped with pool size *n* given by *n* = *m* ^1/2^ = (1/θ)^1/2^ or the nearest integer. Of course, the value of *n* has to be viable in terms of the detection tests. As the maximum value of pooled size^1^ can be 64, *m* can be 4096. The minimum value of *L* is only 128 tests, instead of doing 4096 tests.

This gives an effective way to apply pooling tests with the pooled size determined by an earlier detection rate. Reducing the number of tests would use a smaller number of test kits and test a large number of people faster. This is important when the tests are a mandatory arrangement with the testing fee^2^ paid by the government.
